# Comparative analysis of cytokine profiles of glaucomatous tears and aqueous humour reveals potential biomarkers for trabeculectomy complications

**DOI:** 10.1002/2211-5463.12637

**Published:** 2019-04-23

**Authors:** Éva Csősz, Eszter Deák, Noémi Tóth, Carlo Enrico Traverso, Adrienne Csutak, József Tőzsér

**Affiliations:** ^1^ Biomarker Research Group Department of Biochemistry and Molecular Biology Faculty of Medicine University of Debrecen Hungary; ^2^ Proteomics Core Facility Department of Biochemistry and Molecular Biology Faculty of Medicine University of Debrecen Hungary; ^3^ Department of Ophthalmology Faculty of Medicine University of Debrecen Hungary; ^4^ Clinica Oculistica DiNOGMI University of Genoa and IRCCS Ospedale Policlinico San Martino Genova Italy

**Keywords:** aqueous humour, biomarker, cytokine profile, glaucoma, tear, trabeculectomy

## Abstract

Glaucoma is a multifactorial neurodegenerative disease that causes impaired vision and, in advanced cases, blindness. The increasing prevalence of glaucoma due to an ageing population has necessitated the identification of suitable biomarkers for the early detection of the disease. Aqueous humour (AH) has been proposed as a source of biomarkers, but it can only be collected using a minor, yet invasive surgical intervention. Tears, however, are constantly available and can be collected any time via noninvasive methods. In order to examine the utility of tear as a surrogate for aqueous humour in biomarker development, we compared the levels of 27 cytokines and chemokines in paired samples of tear and aqueous humour using a Luminex multiplex immunobead‐based technique. Significantly higher levels of cytokines in tear compared to aqueous humour were detected suggesting that tear and aqueous humour are not identical in terms of inflammation response. Furthermore, the levels of IFN‐γ, GM‐CSF and IL‐5 in tear were significantly lower in patients who developed complications after one year, but no statistically significant changes in cytokine levels were observed in aqueous humour. These three molecules may have potential as predictive biomarkers for the appearance of late flap‐related complications of trabeculectomy.

**Abbreviations**
AH: aqueous humour
bFGF: fibroblast growth factor
CV: coefficient of variation
ELISA: enzyme‐linked immunosorbent assay
G‐CSF: granulocyte colony‐stimulating factor
GM‐CSF: granulocyte‐macrophage colony‐stimulating factor
IFN: interferon
IL: interleukin
IOP: intraocular pressure
IP‐10: interferon gamma‐induced protein 10
LC‐MS: liquid chromatography coupled mass spectrometry
MCP‐1: monocyte chemoattractant protein 1
MIP: macrophage inflammatory proteins
MMC: mitomycin C
PACG: primary angle‐closure glaucoma
PDGF: platelet‐derived growth factor
POAG: primary open‐angle glaucoma
RANTES: regulated on activation, normal T cell expressed and secreted
TNF: tumour necrosis factor
VEGF: vascular endothelial growth factor

Glaucoma is a multifactorial neurodegenerative disease affecting the optic nerve, leading to impaired vision and, in advanced cases, blindness 1. There are different types of glaucoma, primarily categorized by the iridocorneal angle. Both the open‐angle glaucoma and the angle‐closure glaucoma can be divided into primary and secondary forms, and can also be classified as acute or chronic 2. In glaucoma, neuropathy of the optic nerve and the loss of retinal ganglion cells can be observed, resulting in atrophy of the optic nerve and impairment of visual functions, leading to blindness 3, 4. Taking into consideration the increasing prevalence of glaucoma due to an ageing demographics of modern society, it is crucial to find suitable biomarkers for the early detection of the disease 5. Several studies were carried out to investigate proteins related to the pathophysiological changes observed in glaucoma, most of them focusing on the analysis of the retina, optic nerve, trabecular meshwork and aqueous humour (AH). There were also studies conducted on blood samples, while only few used tear fluid 6, 7, 8, 9, 10.

Using various proteomic methods such as LC‐MS‐based analyses and immunological methods, hundreds of proteins characteristic of glaucoma were identified. Most of these specimens were obtained from the retina and the trabecular meshwork by highly invasive techniques 9, 10, 11, 12, 13, 14. Biological fluids are often used instead of tissue samples for biomarker studies 15. As such, AH is frequently analysed in the examination of glaucoma as a surrogate for ocular biological fluids 9. AH is the product of the ciliary body and is drained from the eye through the trabecular meshwork and the uveoscleral pathway. Proteomic analyses of AH samples from patients with glaucoma revealed important proteins involved in metabolism, inflammatory response and antioxidant defence 9, 16. Extensive studies analysing the amount of inflammatory cytokines revealed higher levels of pro‐inflammatory cytokines in the AH originating from patients with glaucoma, as compared to controls 17, 18, 19, 20, 21.

Despite the usefulness of AH as a source of biomarkers of trabecular meshwork and retinal ganglion cell damage, the invasive collection of this biological fluid prevents its application for the purpose of screening or follow‐ups. On the other hand, tear fluid provides a better alternative, given its availability and ease of collection along with a composition of glaucoma‐related proteins traced directly to the AH. Tears are a valuable biological fluid, widely studied in eye diseases for the purpose of identifying potential biomarkers for eye‐related and systemic diseases 15. In glaucoma, only few studies used tear fluid as a biological source 22, and with proteomic analyses, 27 proteins were identified as potential glaucoma biomarkers, mainly for primary open‐angle glaucoma (POAG) 12. The pro‐ and anti‐inflammatory cytokines were also studied in tear fluid, showing a shifted balance towards the pro‐inflammatory state in glaucoma upon topical medication 23.

Multiplex immunobead‐based assays are often used to examine the level of multiple cytokine and chemokine molecules in a single sample. This is very beneficial when analysing certain bodily fluids that can be collected only in small amounts, as in the case of tear and AH, respectively 24, 25. In this pilot study, we compared the concentrations of 27 cytokine and chemokine molecules in the tear and AH samples originating from the same patient with glaucoma, using a commercially available multiplex immunobead kit. Our aim was to determine whether the readily available tear fluid can replace the use of AH in studying patients with glaucoma following trabeculectomy.

## Results and Discussion

Multiplex immunobead assays provide a versatile tool for the analysis of bodily fluids with low volume. The application of antibodies provides high specificity, while the Luminex beads offer the possibility of multiplexing, allowing for the analysis of multiple analytes in one sample 26. This technique was used in the form of a validated, commercially available kit for the measurement of the concentration of 27 cytokine and chemokine molecules in tear and AH samples originating from patients with glaucoma, who underwent trabeculectomy in order to lower their intraocular pressure. All the 27 analytes were detected in both sample types; however, in cases where the amount of the studied molecule was under the limit of detection (Table S1), the number 0 was assigned for the concentration of the analyte. The most dilute standards from the calibration curve (S8) had high CV% in case of most of the analytes, indicating that the kit cannot reliably measure the concentration of the individual proteins in very dilute samples (Table S1).

### The analysis of tear

All of the examined cytokines, except for IL2 and IL10, were present in at least 90% of the tear samples. IL2 could be detected only in 45% of the samples, while IL10 was present in 85% of the samples (Table S2). Considering that we did not include healthy control because our aim was to compare the two types of samples originating from the same patient, first we compared our results to data available in the literature.

Comparing our results to those obtained by Martinez‐de‐la‐Casa *et al*. 27 regarding the concentration of the same 27 cytokines and chemokines on control and glaucoma subjects using the same sample collection procedure, we can conclude that the levels of eotaxin, G‐CSF, IL‐12, IL‐13, IL‐15, IL‐1β, IL‐4, IL‐7 and MIP1α were in the same range and were very similar in the two studies. A similar trend in the levels of bFGF, GM‐CSF, IFN‐γ, IL‐10, IL‐17, IL‐5, MCP1, PDGF‐BB, TNFα and VEGF was observed as compared to controls in the two studies; however, in our case, the levels of these cytokines were even higher. The levels of IL‐1Ra, IL‐2, IL‐6, IL‐8, IP‐10, MIP1β and RANTES were lower in our study as compared to the control tears from healthy subjects published by Martinez‐de‐la‐Casa *et al*. (Table S3) 27. However, when we compared the cytokine levels measured in our samples to the results reported by Mrugacz et.al. examining physiological tear with ELISA method 28, the levels of IL‐6, TNFα and IL‐17 were much higher in our samples. A similar phenomenon was observed when we compared our data to those obtained by Gupta et al. 29 examining eye‐drop naive patients with newly diagnosed POAG compared to controls using a different sample collection procedure and a different method than ours.

It should be noted that it is indeed difficult to compare results obtained by different studies, as the application of different analytical strategies hinders the proper comparison of results between studies. For tear analysis, Gupta et al. and Mrugacz et.al. have used ELISA methods to examine the cytokine levels. Their results are comparable to each other but display magnitude lower values compared to our results and the results obtained by Martinez de la Casa *et al*. 27, 28, 29. The higher level of some cytokines observed in our study compared to the values from the literature might indicate a more pronounced pro‐inflammatory condition, which might be due to the fact that the patients recruited into our study had a more advanced phase of glaucoma, finally succumbing to trabeculectomy.

Another factor that can influence tear composition is the method of collection. Depending on the procedure, significantly higher levels of pro‐inflammatory cytokines were observed in basal tears as compared to reflex tears 30. In our study, basal tear was collected by the same person throughout the study, and the presence of ocular surface disease or autoimmune disease was regarded as exclusion criteria. It is important to note that neither the tear collection nor the analysis method applied by Gupta et al. was the same as the ones used in this study and by Martinez‐de‐la‐Casa et. al., and the patients with glaucoma recruited in these two later studies were eye‐drop users 27, 29. According to the currently used treatment protocols, eye‐drops are widely used in the therapy of glaucoma. Evidence from the literature shows that the administration of eye‐drops can distort the level of cytokines in tears, leading to higher pro‐inflammatory cytokine concentrations, in agreement with the findings from our study 23, 31.

Gender and the type of glaucoma were not associated with a statistically significant difference on the cytokine levels, tear production rate and tear protein concentration in any of the studied groups. The group of patients using three or more different eye‐drops was compared to the group using less than three eye‐drops, but we could not find any statistically significant difference (Table S4). Based on these data, we think that the administration of different drugs does not modulate the level of the analysed cytokines, tear protein concentration or tear production rate, at least in case of patients examined in this study.

The IOP of the patients and the presence of complications following trabeculectomy were monitored, including infection, blebitis, endophthalmitis, reduced visual acuity, flap‐related complications, choroidal ablation, and bleb failure leading to blocked AH filtration to the subconjunctival space.

**Figure 1:**
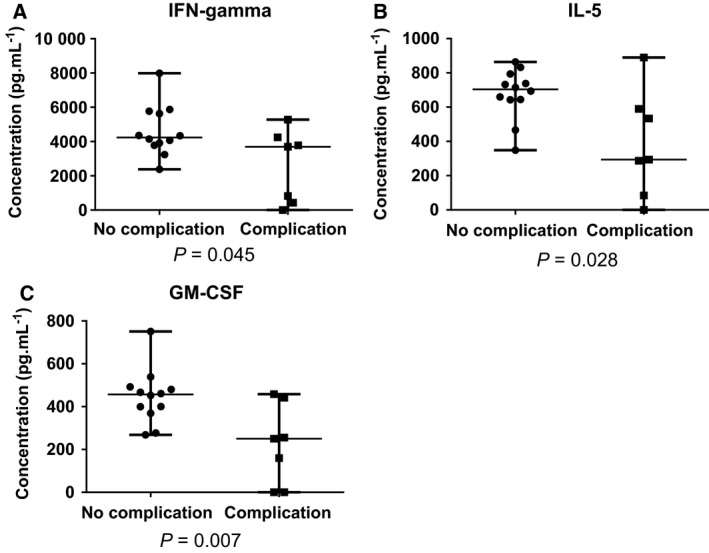
Concentrations of cytokines with statistically significant differences between the groups with complications and no complication. The concentration (pg·mL^−1^) of IFN‐gamma (A), IL‐5 (B) and GM‐CSF (C) in the two groups is plotted. Below each plot, the *P* values obtained by the utilization of nonparametric Mann–Whitney *U*‐test are indicated.

We collected ophthalmological data for 19 out of 20 recruited patients, and we examined the effect of the presence of complications on the level of tear cytokines, tear total protein concentration and tear production rate. In case of three proteins, statistically significant differences could be observed. The concentration of IFN‐γ was higher in the group of patients without complication, and the same tendency was observed in case of GM‐CSF and IL‐5, as well (Fig. 1).

IFN‐γ is secreted by T helper cells, cytotoxic T cells and natural killer cells and has antiviral, immunoregulatory and antitumour properties 32. The increase of IFN‐γ may inhibit collagen synthesis and lead to delayed epidermal wound healing 33, 34. According to another study, the application of mesenchymal stem cells following chemical injury of the cornea reduced IFN‐γ levels and thus reduces the corneal inflammation and neovascularization resulting in wound healing with less epithelial defects 35.

IL‐5 is mainly produced by Th2‐type helper T cells and was shown to have an important role in the activation, proliferation and survival of eosinophils and the differentiation of B cells 36. It was observed in mice overexpressing IL‐5 that the wound healing is delayed and re‐epithelization is impaired 37.

GM‐CSF is a pleiotropic molecule activating granulocyte and macrophage cell lineages, with an important role in wound healing through the induction of keratinocyte proliferation and promoting migration of epithelial cells 38. Recombinant GM‐CSF can be applied to help the wound healing process 38. It was shown that GM‐CSF plays an important role in the early, so‐called acute inflammatory phase of ocular wound healing 39. With the administration of recombinant human GM‐CSF, it was demonstrated that GM‐CSF has no effect on cell proliferation but on cell migration 40.

According to our data, ocular complications are associated with reduced levels of each observed cytokine. However, according to the scientific literature, faster wound healing, which might be one reason for bleb failure, would require increased levels of these factors. It should be noted that none of the studies involving IL‐5, IFN‐γ and GM‐CSF were carried out on tear samples. The phenomena leading to bleb failure are not known in detail, and most probably, multiple, more complex mechanisms take part in the regulation of the process.

Despite the fact that our results observed in human tear contradict the results observed by others in skin or in rodent ocular wound healing models, IL‐5, IFN‐γ and GM‐CSF can serve as a good starting point for further biomarker studies and verifications.

### The analysis of aqueous humour

Aqueous humour was next analysed in the same manner as tear. More than half of cytokines detected were present in more than 90% of the samples. However, IL‐1b, IL‐7, IL‐8, IL‐9, IL‐10, G‐CSF and IFN‐γ were present in less than 50% of the samples. IL‐2, IL‐4, IL‐5, IL‐13, bFGF and MIP1α were present in 70–85% of the samples.

Next, we compared our data to data acquired from the analysis of human AH removed during cataract surgery by Sharma *et al*., POAG and cataract samples by Duvesh *et al*. and control and PEX samples by Garweg *et al*. (Table S3) 24, 41, 42. We found that the level of bFGF, G‐CSF, IL‐1Ra, IL‐6 and RANTES was higher in our samples; the level of IL8, MIP1α, MIP1β and eotaxin was higher in the study carried out by Sharma et. al.; and the MCP1 levels were in the same range in both studies 24. Based on other studies, where similar examination methods were used to those applied by our group, the levels of eotaxin, GM‐CSF, IL‐1β, IL‐6, IL‐8, IL‐9, IL‐10, IP‐10, MCP1, MIP1α and VEGF were in the same range, and the level of IL‐12, IL‐4 and MIP1β was lower in our study. At the same time, the levels of bFGF, G‐CSF, IL‐1ra, IL‐2, IL‐5, IL‐7, IL‐13, IL‐15, IL‐17 and TNFα were higher in our study compared to the studies carried out by Duvesh *et al*. and Garweg *et al*. (Table S3) 41, 42.

The effect of complication, gender and type of glaucoma on the cytokine levels was examined but no statistically significant difference could be found between any of the studied groups (data not shown).

### Comparative analysis

**Figure 2:**
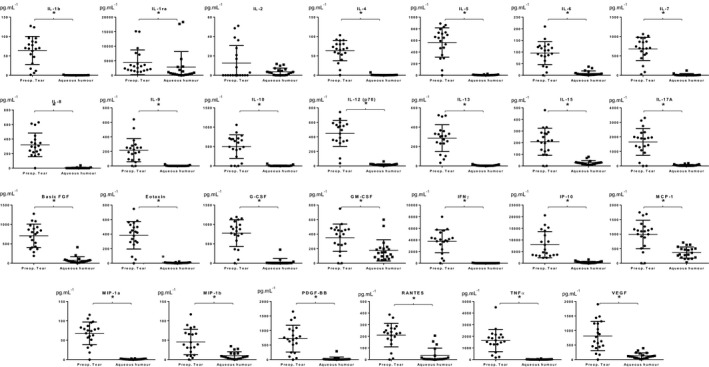
Concentrations of 27 cytokines and chemokines in preoperative tear and aqueous humour samples from patients with glaucoma. The ‘*x*’ axis shows the sample type, while ‘*y*’ axis shows the concentration of the analyte in pg·mL^−1^. For statistical analysis, paired nonparametric Wilcoxon test was used, and * indicates statistically significant (*P* < 0.05) difference.

Tear, as a noninvasively collectable bodily fluid, is constantly available and an important resource for follow‐up studies. Different studies have examined the composition of tears and of aqueous humour in glaucoma and other eye diseases, but no previous comparative study examining the cytokine levels in tears and aqueous humour was carried out. After critical examination of the data obtained for tear and AH, we have compared the level of cytokines between these two sample types. All studied cytokines and chemokine molecules, except IL‐2, showed statistically significantly higher levels in tears compared to AH (Fig. 2, Table 1).

**Table 1:** Concentrations of cytokines and chemokines in preoperative tear and aqueous humour samples by multiplex bead immunoassay. Data are expressed as mean (pg·mL^−1^) ± SEM. The concentrations were calculated based on the recorded 8 point calibration curves and applied dilutions shown in Table S1. Statistical significance between groups was determined using paired nonparametric Wilcoxon test. The *P* values in bold indicate statistically significant (*P* < 0.05) values. SEM, standard error of the means

Cytokine	Preoperative tear	Aqueous humour	*P* value
bFGF	725.48 ± 114.51	75.86 ± 26.13	**0.0001**
Eotaxin	388.04 ± 71.72^a^	5.95 ± 1.99^a^	**0.0001**
G‐CSF	774.46 ± 131.24^a^	25.58 ± 20.46	**0.0001**
GM‐CSF	358.06 ± 73.92	174.49 ± 49.2^a^	**0.0008**
IFN‐g	3762.96 ± 680.68	6.75 ± 4.84	**0.0001**
IL‐10	497.45 ± 116.38	5.96 ± 4.7^a^	**0.0004**
IL‐12	450.22 ± 72.97^a^	16.98 ± 4.63	**0.0003**
IL‐13	293.49 ± 52.67	4.90 ± 1.93	**0.0001**
IL‐15	211.71 ± 43.68^a^	26.80 ± 5.69	**0.0001**
IL‐17	1670.93 ± 352.26	47.26 ± 20.82	**0.0001**
IL‐1b	64.18 ± 13.65^a^	0.23 ± 0.15^a^	**0.0001**
IL‐1ra	3236.83 ± 824.22	1113.62 ± 504.18	**0.0031**
IL‐2	12.77 ± 7.47	6.58 ± 3.18	0.4380
IL‐4	63.94 ± 9.91^a^	0.72 ± 0.36	**0.0001**
IL‐5	553.52 ± 102.4	4.82 ± 2.36	**0.0001**
IL‐6	94.64 ± 17.75	43.77 ± 31.11^a^	**0.0038**
IL‐7	686.74 ± 109.95^a^	9.85 ± 7.47	**0.0001**
IL‐8	315.37 ± 55.32^a^	2.74 ± 2.33^a^	**0.0002**
IL‐9	210.35 ± 57.31^a^	2.13 ± 1.67^a^	**0.0004**
IP‐10	7816.74 ± 2268.70	383.96 ± 157.47^a^	**0.0001**
MCP‐1	1004.36 ± 179.03	365.66 ± 82.18^a^	**0.0004**
MIP‐1a	67.12 ± 11.05^a^	1.05 ± 0.39^a^	**0.0001**
MIP‐1b	45.41 ± 13.08	9.98 ± 3.95	**0.0007**
PDGF‐BB	707.12 ± 173.6	22.71 ± 14.34	**0.0001**
RANTES	210.20 ± 35.7	34.91 ± 22.33	**0.0001**
TNF‐a	1466.11 ± 265.38	13.49 ± 6.78	**0.0001**
VEGF	805.66 ± 200.44	130.83 ± 37.16^a^	**0.0001**

a Indicate values which are in the same range with data obtained by other studies.

Based on our data, we can conclude that tear and aqueous humour are not identical from the inflammatory point of view and tear cannot substitute for aqueous humour for diagnostic purposes. Most probably, the levels of cytokines in the two sample types are controlled by different mechanisms. The higher cytokine levels in tear compared to aqueous humour might reflect not only the glaucoma‐related, but also the eye‐drop‐ or other factors‐induced pro‐inflammatory conditions, as well. A limitation of the study is the relatively low number of the participants recruited. Larger studies are needed to validate our findings and also to be able to do a proper patient stratification based on the drugs used.

## Materials and methods

### Subjects and sample collection

All participants recruited into this study were patients of the Department of Ophthalmology, Faculty of Medicine, University of Debrecen. The participants provided written informed consent for tear and aqueous humour collection in accordance with the Declaration of Helsinki. The study was approved by the Ethical Committee of the University of Debrecen (approval number: 4234‐2014). All patients underwent trabeculectomy surgery to reduce intraocular pressure. Exclusion criteria were the presence of autoimmune and/or ocular surface diseases and previous history of ocular surgery within 6 months. For the comparison of the tear and aqueous humour, 20 patients (11 male and 9 female; mean age: 58.8 ± 14.8) with glaucoma [eight patients with primary angle‐closure glaucoma (PACG) and 12 patients with POAG] were recruited and the tears and aqueous humour were collected. Basal tear was collected only once for 2 min from patients except G002 and G093 right before the trabeculectomy. In case of patients G002 and G093, sample collection was not possible on the day of trabeculectomy; in their case, a previously collected tear sample was used. These cases are marked with * in Table S2. The nontraumatic tear collection was carried out before the trabeculectomy, using sterile glass capillary tubes (VWR Ltd, Hungary) from the lateral inferior meniscus without local anaesthesia or stimulation 43. Tear samples were collected for 2 min, and then promptly transferred to 0.2‐mL PCR tubes and centrifuged on 4 °C (264 ***g***) for 10 min, and the supernatants were then deep‐frozen and stored at −70 °C until analysis. The AH samples were collected during trabeculectomy surgery through a limbal paracentesis by the same operator using sterile glass capillary. Care was taken to prevent blood and intraocular tissue contamination. The samples were expelled from the capillaries to 0.2‐mL PCR tubes and processed in an identical way as tears. Protein concentration of tear and aqueous humour samples was determined using the Bradford method 44.

### Multiplex analysis of cytokines in tear and aqueous humour samples

The concentration of the interleukins IL‐1β, IL‐1Ra, IL‐2, IL‐4, IL‐5, IL‐6, IL‐7, IL‐8, IL‐9, IL‐10, IL‐12p70, IL‐13, IL‐15, IL‐17, eotaxin, basic fibroblast growth factor (bFGF), granulocyte colony‐stimulating factor (G‐CSF), granulocyte‐macrophage colony‐stimulating factor (GM‐CSF), interferon (IFN)‐γ, interferon gamma‐induced protein 10 (IP‐10), monocyte chemoattractant protein 1 (MCP1), macrophage inflammatory proteins MIP1α and MIP1β, platelet‐derived growth factor (PDGF‐BB), regulated on activation, normal T cell expressed and secreted (RANTES), tumour necrosis factor α (TNFα) and vascular endothelial growth factor (VEGF) was analysed using a multiplex immunobead system based on xMAP technology (Luminex, Austin, TX, USA). The 27‐plex Bio‐Plex kit (Bio‐Rad Laboratories, Hercules, CA, USA) was utilized strictly adhering to the manufacturer's instructions. The tear and aqueous humour samples were diluted. Based on previous experiments, 1 : 25 dilution was applied for tears and 1 : 2 dilution for AH samples, where the amount of the AH was sufficient. In cases where the amount of the AH collected was not enough, the dilutions indicated in Table S1 were used. Two technical replicates were used in each case; however, in case of G041 and G107 the tear sample amount was enough only for one replicate (samples marked with # in Table S2).

Fluorescence intensity data were recorded and analysed using the bio‐plex manager software (version 4.1.1.; Bio‐Rad Laboratories). Concentrations of the 27 cytokines and chemokines were calculated based on the recorded calibration curve and the dilution factor used. For curve fitting, logistic regression was applied.

### Statistical analysis

Statistical analysis was performed using spss 25.0 (IBM, Armonk, NY, USA) for Windows. Nonparametric Mann–Whitney U‐test was used to compare the protein concentrations among groups. For the comparison of tear and aqueous humour samples originating from the same patient, paired nonparametric Wilcoxon test was used. All data with p<0.05 are defined as significantly different.

## Conclusion

In our study, we compared the levels of 27 cytokines in tear and AH originating from patients with glaucoma who underwent trabeculectomy. Our findings are largely in agreement with the data in the scientific literature; however, in the case of some cytokines differences were detected. According to our data, tear and AH are not identical from the inflammatory point of view; significantly higher levels of cytokines were found in tear compared to AH.

The concentrations of three tear proteins, IFN‐γ, GM‐CSF and IL‐5, were associated with the presence of complications. However, more patients have to be recruited to be able to identify/verify these proteins as potential risk factors for the occurrence of flap‐related complications following trabeculectomy.

Our data highlight the promising potential of this continuously available, easy‐to‐collect body fluid for dynamic testing, making it worth exploring as an alternative to samples collectable by invasive methods.

## Conflict of interest

The authors declare no conflict of interest.

## Author contributions

ÉC and AC conceptualized the study. ÉC and NT curated the data. ÉC and JT acquired funding. ÉC, ED and NT contributed to methodology. ÉC, JT and AC provided resources. ÉC visualized the data. ÉC wrote the original draft of the manuscript. CET, JT and AC reviewed and edited the manuscript.

## Supporting information


**Table S1.** Raw data obtained by the multiplex analysis of cytokines and chemokines in preoperative tear and aqueous humour samples from patients with glaucoma.

**Table S2.** Clinical features and sample characteristics of patients with glaucoma. In case of each patient recruited the type of glaucoma, age, gender, number of preoperatively used drugs, the tear flow rate, collected tear volume and tear protein concentration, the collected AH volume and AH protein concentration and the presence of postoperative complications are presented along with the concentrations of the 27 cytokines and chemokines.

**Table S3.** Comparison of the concentrations of cytokines and chemokines measured in tears and aqueous humour to data published in the scientific literature.

**Table S4.** The effect of gender, type of glaucoma and the number of eye drops administrated on the concentration of cytokines and chemokines. The number of cases in each group, the mean rank, the sum of ranks and the p values given by the nonparametric Manny‐Whitney U‐test are shown in case of each analyte.
